# Effect of the Order-Disorder Transition on the Electronic Structure and Physical Properties of Layered CuCrS_2_

**DOI:** 10.3390/ma14112729

**Published:** 2021-05-21

**Authors:** Evgeniy V. Korotaev, Mikhail M. Syrokvashin, Irina Yu. Filatova, Aleksandr V. Sotnikov

**Affiliations:** Nikolaev Institute of Inorganic Chemistry, Siberian Branch, Russian Academy of Sciences, 630090 Novosibirsk, Russia; syrokvashin@niic.nsc.ru (M.M.S.); rare@niic.nsc.ru (I.Y.F.); sotnikov@niic.nsc.ru (A.V.S.)

**Keywords:** layered copper-chromium disulfide, Seebeck coefficient, electrical resistivity, order-disorder transition, DSC, DFT

## Abstract

The work reports a comprehensive study of the Seebeck coefficient, electrical resistivity and heat capacity of CuCrS_2_ in a wide temperature range of 100–740 K. It was shown that the value of the Seebeck coefficient is significantly affected by the sample treatment procedure. The order-to-disorder (ODT) phase transition was found to cause a metal-insulator transition (MIT). It was established that the ODT diminishes the Seebeck coefficient at high temperatures (T > 700 K). The DFT calculations of the CuCrS_2_ electronic structure showed that the localization of copper atoms in octahedral sites makes the band gap vanish due to the MIT. The decrease of CuCrS_2_ electrical resistivity in the ODT temperature region corresponds to the MIT.

## 1. Introduction

Waste energy harvesting is a pressing problem of modern highly efficient industries. Waste energy accounts for about a half of the total energy consumption. Therefore, new types of thermoelectric materials for direct conversion of waste heat into electric energy have been developed [1,2]. The modern materials science is focused on material nanostructuring. Nanostructured materials usually combine high electronic conductivity and thermal resistivity due to the difference between their electron and phonon mean free paths. Material nanostructuring typically requires a special high cost layer-by-layer processing or synthesis techniques. Natural nanostructured materials are of special interest due to the self-assembly exhibited by these materials. Layered transition metal dichalcogenides MX_2_ (M is a transition metal; X = S, Se, Te) can be considered as nanostructured materials of natural origin. These compounds are formed by alternating metal and chalcogenide layers. The unstable MX_2_-layers can be stabilized by the intercalation of metal atoms between dichalcogenide layers [3,4,5,6,7,8,9]. Intercalation or cationic substitution affect significantly the physical properties of MX_2_ based compounds [8,9,10,11,12]. Copper-chromium disulfide CuCrS_2_ is considered to be a promising functional material for electronic devices. Undoped copper-chromium disulfide and CuCrS_2_ based solid solutions exhibit a wide range of physical properties such as thermoelectricity [9,10,11,12,13,14,15,16], ionic conductivity [17,18,19,20,21] and various magnetic properties [22,23,24]. Thanks to the combination of ionic conductivity and thermoelectric properties (the Seebeck coefficient, electric and thermal conductivities, ZT) demonstrated by CuCrS_2_ and similar layered transition metal dichalcogenides MCrX_2_ (M = Cu, Ag; X = S, Se), these compounds can be considered phonon glasses. Phonon glasses usually have a high value of the Seebeck coefficient due to the “fixed” glass matrix and low thermal conductivity as a result of phonon scattering on mobile cations [8,9,13,25,26]. The ionic conductivity of MCrX_2_ is caused by the redistribution of mobile metal atoms over crystallographic sites in the MX_2_ sublattice. As the temperature increases, the mobile metal atoms begin to occupy the sites that were unoccupied in the room temperature region. As a result, the MCrX_2_ conductivity and the order-disorder phase transition (ODT) are increased. Note that CuCrS_2_ structure does not significantly changes before and after ODT phase transition [27], in contrast the traditional to SnSe- and GeTe-based thermoelectric materials [28,29], where phase transition is accompanied by the spatial group changes. Hence, the similarity of CuCrS_2_ and Se/Te-based systems is that both demonstrate phase transitions. The difference is that ODT phase transition in CuCrS_2_ does not significantly affect the crystallographic structure [27]. Both Se/Te-based systems materials were reported to have a promising ZT values of ~1.5. However, CuCS_2_ was also reported to have high ZT values of ~2 [14]. Thus, this fact makes it promising to study the thermoelectric properties of CuCrS_2_ and CuCrS_2_-based materials. The ODT increases structural disorder and, therefore, enhances phonon scattering and suppresses the lattice thermal conductivity. Thus, one can conclude that thermoelectric properties of MCrX_2_ can be significantly affected by the ODT phase transition [26]. However, most of the reported data concerning thermoelectric properties of CuCrS_2_ and CuCrS_2_ based solid solutions were carried out in the temperature range below the ODT [11,12,14]. Thus, the ODT influence on the CuCrS_2_ thermoelectric properties has not been discussed yet. Note that the redistribution of mobile metal atoms over the crystallographic sites also affects the CuCrS_2_ electronic structure. The electronic structure of valence and conduction bands is the key aspect when interpreting and predicting the character of thermoelectric properties [9,11]. Therefore, DFT calculations of DOS distribution were carried out to interpret the temperature dependence of the CuCrS_2_ Seebeck coefficient in the ODT region.

## 2. Experimental

**Figure 1:**
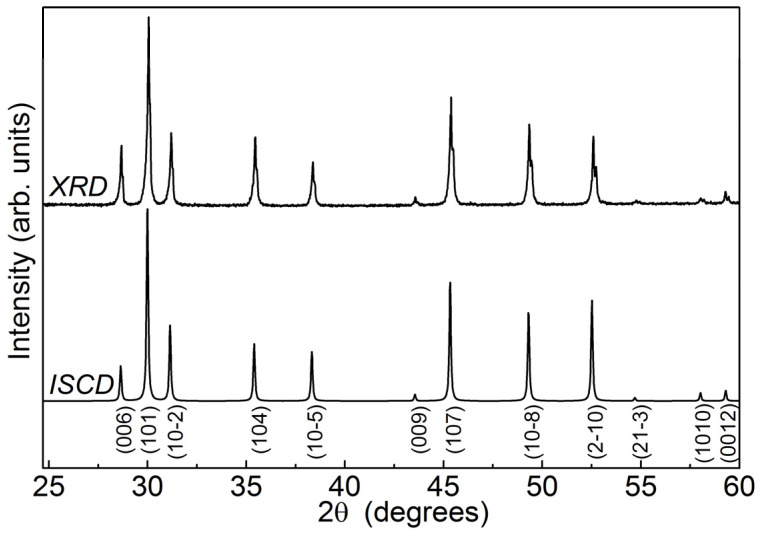
Powder diffraction pattern of the synthesized CuCrS_2_ sample (XRD) and reference data (ICSD).

The initial CuCrS_2_ powder sample was synthesized from copper and chromium oxides (CuO, Cr_2_O_3_) with a purity of 99.99% (Millipore Sigma, St. Louis, MO, USA; MSE Supplies LLC, Tucson, AZ, USA). A mixture of initial metal oxides in a horizontal glassy carbon boat was placed in a quartz reactor. The air was removed from the reaction volume by argon and ammonium rhodanide (NH_4_SCN) decomposition products of gas flow. The reaction mixture was heated to 1050 °C and ground for several times during the synthesis. The completeness of sulfidization was controlled by powder X-ray diffraction (XRD) and by weighing the sample. The XRD experiment was carried out using non-monochromatic CuKα-radiation (Shimadzu XRD 7000S diffractometer, Shimadzu Corporation, Kyoto, Japan). The XRD pattern of the synthesized CuCrS_2_ sample is shown in Figure 1. The XRD pattern indicates that the synthesized sample is composed of a single phase corresponding to the rhombohedral space group (*R3m*). The positions of diffraction peaks and intensity ratios are in good agreement with the data of the Inorganic Crystal Structure Database (denoted “ICSD” in Figure 1) [30]. The calculated unit cell parameters *a* = 3.480(4) and *c* = 18.689(5) Å correlate well with previously reported and reference data [11,27,30].

**Figure 2:**
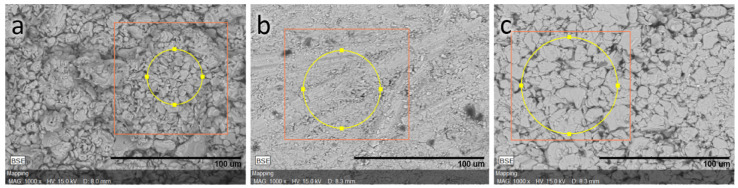
SEM images of CuCrS_2_ samples: powder (**a**), vacuum compressed (**b**) and argon atmosphere treated samples (**c**).

The ceramic samples were prepared using the synthesized CuCrS_2_ powder sample in the course of a two-step procedure. At the first step, the samples were subjected to 10 MPa cylindrical compression at room temperature in air. At the second step, the compressed samples were treated under different conditions (Table 1). The composition of compressed samples (Table 2) was analyzed by scanning electron microscopy (Hitachi TM3000, Tokyo, Japan, microscope equipped with a Bruker EDS QUANTAX 70 analyzer, Billerica, MA, USA). The backscattered electron (BSE) images are shown in Figure 2. The SEM images were made with a 1000× magnification. The elemental composition of the studied samples correlates well with the theoretical reference concentration. The EDS analysis was performed with an accuracy of ~1%. The SEM images indicate that the sample’s density increases in the series powder→argon treated sample→vacuum treated sample. The most homogeneous surface was observed for the vacuum treated ceramic sample (Figure 2). This fact agrees well with the density measurements of ceramic samples (Table 1).

The temperature dependence of the Seebeck coefficient was measured in a rarefied 5 Torr helium atmosphere with samples placed between two copper contact pads. The 5 °C temperature gradient between the copper pads was maintained using a Thermodat-13K5 temperature controller (LLC RPE Control Systems, Perm, Russia). The thermoelectric power between the copper pads was measured using a Keysight 34465A digital voltmeter (Keysight Technologies, Santa-Rosa, CA, USA). The electrical resistivity was measured as the resistance between the copper pads. In this case, the temperature difference between the pads did not exceed 0.5 K.

The thermal effects accompanying the ODT phase transition were studied on a DSC-500 differential scanning calorimeter (LLC Specpribor, Samara, Russia). A 20 mg sample was measured in an open aluminum crucible with a heating rate of 10 °C/min in a 50 mL/min argon flow.

The valence band partial density-of-states (pDOS) distribution was calculated with the BAND package [31] using the generalized gradient approximation (GGA), a standard Slater-type basis set with three basis functions per atomic orbital, one polarization function (TZP) and the Perdew-Burke-Ernzerhof exchange-correlation potential (PBESol-D). The initial atomic coordinates were taken from the ICSD [30]. In the case of copper atoms localized at the octahedral sites of the van der Waals gap, the geometry was optimized using the initial atomic coordinates taken from [27].

## 3. Results and Discussion

The thermoelectric properties of copper-chromium disulfide and CuCrS_2_ based solid solutions are significantly influenced by the synthesis conditions and the sample preparation procedure [11,12,13,14,15,16]. Thus, it is particularly interesting to analyze the dependence of the Seebeck coefficient on the sample treatment procedure.

**Figure 3:**
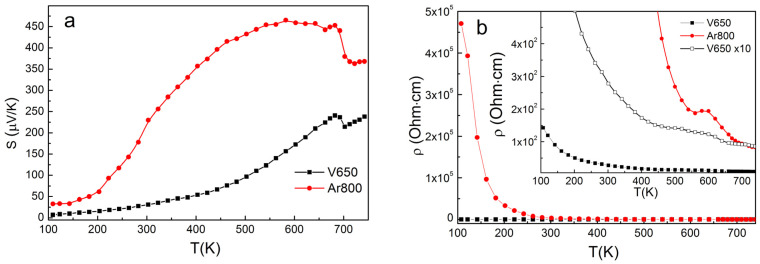
Temperature dependencies of the Seebeck coefficient (**a**) and electrical resistivity (**b**) for compressed CuCrS_2_ samples. The inset shows the enlarged curves.

Figure 3a shows the temperature dependencies of the Seebeck coefficient (S) for compressed CuCrS_2_ samples. The positive sign of S indicates the *p*-type conductivity. This fact is in good agreement with previously reported data [11,12,13,14,15,16]. Note that the treatment procedure does not affect the conductivity type. The largest S value of ~450 µV/K (at T ~550 K) was observed for the argon treated sample at 800 °C (Ar800). The S values measured for the vacuum treated sample at 650 °C (V650) are lower than those measured for the vacuum treated sample. On the other hand, the electrical resistivity for the argon treated sample is one to three orders larger than for the vacuum treated sample (Figure 3b). This can be due to the fact that the argon treated sample has a lower density than the vacuum treated sample (Table 1). At the same time, the electrical resistivity of both samples decreases with temperature, which is typical of semiconductor materials. Thus, annealing the sample in the argon atmosphere can be an efficient procedure to increase the value of the Seebeck coefficient of CuCrS_2_ based materials. The hot vacuum pressing procedure increases the sample density. The sample treatment in vacuum at 650 °C results the optimum Seebeck coefficient value (~200 uV/K), typical for SnSe- and GeTe-based thermoelectric materials [28,29]. Thus, the sample treatment procedure could be used to optimize the Seebeck coefficient value both the cationic substitution of CuCrS_2_-matrix [11].

**Figure 4:**
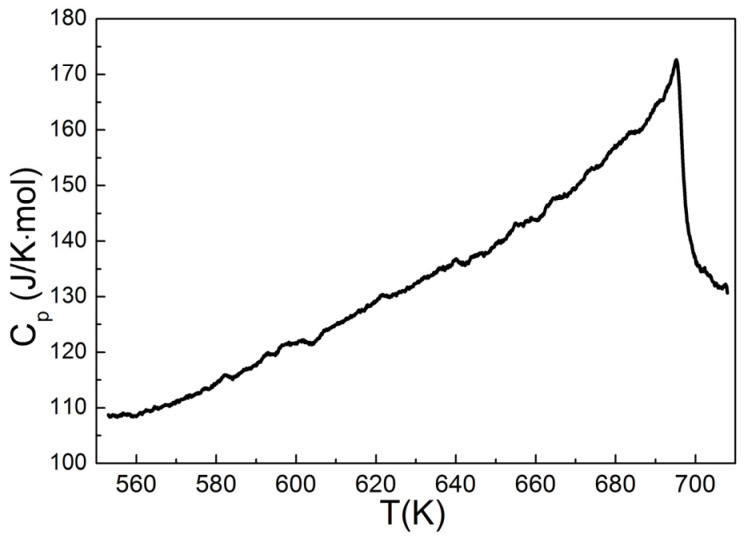
Temperature dependence of heat capacity for CuCrS_2_.

Above 600 K, the Seebeck coefficient and electrical resistivity exhibit similar temperature dependencies for all studied samples. Both S(T) and ρ(T) temperature dependencies exhibits inflection features in the corresponding temperature region at T~600–700 K. This fact correlates well with the data reported in [15]. Note that the presence of inflection is not affected by the treatment procedure and can be therefore related to physical properties of CuCrS_2_. For instance, the order-disorder phase transition (ODT) occurs in the same temperature region [17,18,19,20,21,22,27]. Differential scanning calorimetry (DSC) is the most common experimental technique to study phase transitions. The DSC sensitivity for ODT in similar chalcogenides AgCrS_2_ was reported in [26,32]. However, DSC has not yet been used to study ODT in CuCrS_2_. The temperature dependence of heat capacity is shown in Figure 4. The investigated temperature region exhibits a single peak at 695 K corresponding to an abrupt C_p_ decrease. The shape of the C_p_(T) line is characteristic of the second-order phase transition. Thus, the observed phase transition corresponds to the ODT and agrees well with the previously reported data [26,27,32]. The inflection feature on the S(T) curves lies in the same temperature region as the ODT. Thereby, the decrease of the Seebeck coefficient at T > 700 K is due to copper migration from the “ordered” tetrahedral sites to the “disordered” ones [26,27]. The positions of ρ(T) infection features are shifted to lower temperatures compared to those exhibited by S(T) and C_p_(T) temperature dependences. Note however that inflection features appear on the temperature dependencies of electrical resistivity as a result of ODT [33]. Since thermopower, electrical resistivity and heat capacity temperature dependencies involve different physical processes, the inflection point of these dependencies may occur at different positions. Hence, the observed inflection features on S(T), ρ(T) and C_p_(T) curves are of the same origin and correspond to the order-to-disorder transition.

**Figure 5:**
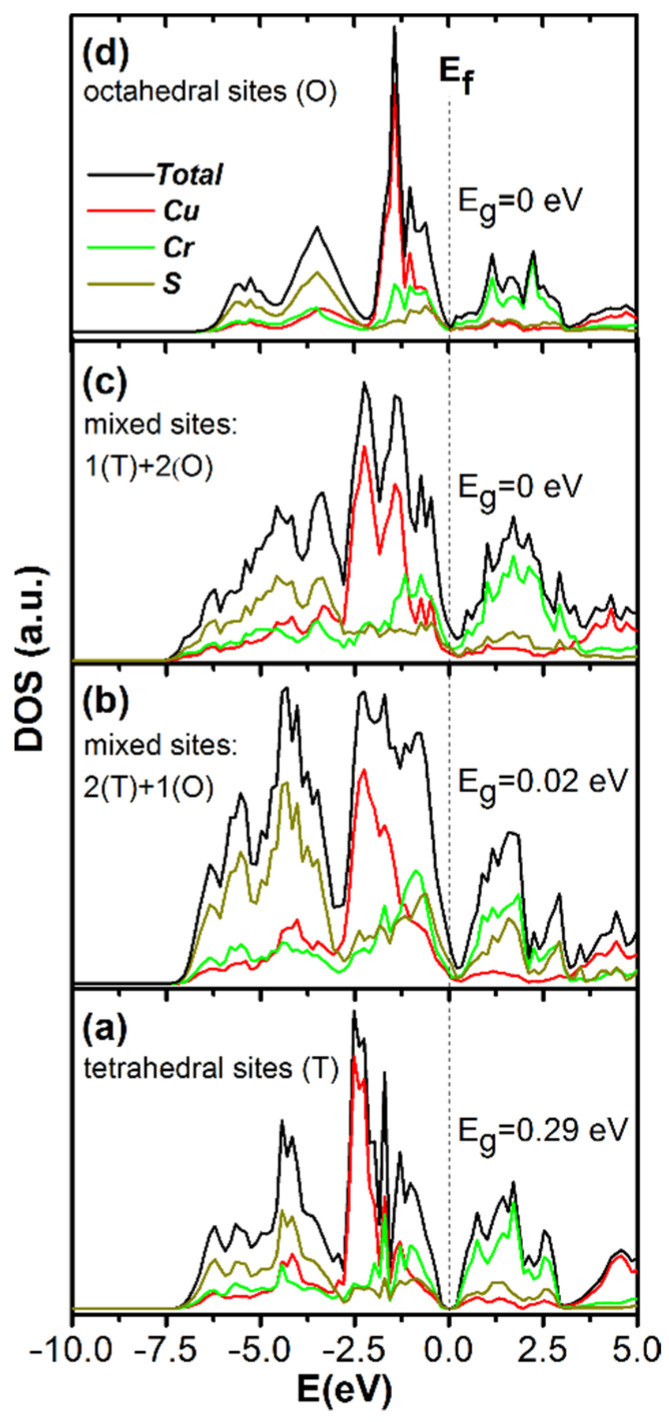
Simulated DOS of CuCrS_2_: copper atoms localized at tetrahedral (**a**), octahedral (**d**) and mixed both tetrahedral and octahedral sites (**b**,**c**).

Different crystallographic sites between the CrS_2_ layers (the van der Waals gap region) are tetrahedral and octahedral *o*-sites. In the ordered state at room temperature, the copper atoms are localized at the tetrahedral sites [9,27,34,35]. The probability of the localization of *o*-sites increases in the ODT temperature region. The redistribution of copper atoms between different sites can affect the CuCrS_2_ electronic structure. It was previously reported that the value of the Seebeck coefficient is significantly affected by the structure of the partial density of states (pDOS) in conduction and valence bands [9,11]. Thus, the ODT influence on the CuCrS_2_ electronic structure is of special interest. Figure 5 shows pDOS distributions of CuCrS_2_ calculated for different copper sites. Figure 5a shows the electronic structure corresponding to the localization of copper atoms at the “ordered” tetrahedral sites. The main contributions of copper and chromium *d*-states are localized near the valence band top at −2.5 and −1 eV below the Fermi level (denoted as “E_f_” in Figure 5), respectively. The main contribution of sulfur *p*-states is localized deeply in the valence band at −4 eV. Note that the most significant contribution to the valence band structure is due to copper states and corresponds to the filled d-electron shell of copper (3*d*^10^ configuration). The conduction band bottom is mainly constituted by chromium *d*-states formed by the unfilled chromium d-shell (3*d*^3^ configuration). The contribution of sulfur and copper states to the structure of the conduction band bottom is smaller. According to the obtained data, the copper-chromium disulfide is a semiconductor with a band gap of ~0.29 eV (denoted as “E_g_” in Figure 5). The calculated pDOS is in good agreement with experimental and calculated data reported in [9,36].

The localization of copper atoms in the octahedral *o*-sites significantly affects the pDOS (Figure 5d). As a result of pDOS redistribution, the valence band narrows while the valence band edge is shifted from −7 to −6 eV. The most significant contribution to the structure of the CuCrS_2_ valence band is still related to the copper states.

However, the contribution of copper states is shifted to the high energy region and is localized at −1.5 eV. The character of sulfur and chromium pDOS distribution is generally preserved. Nevertheless, the main contribution of sulfur is slightly shifted to the high energy region as a result of the valence band narrowing. Note that the intensity of the total DOS at the conduction band bottom is lower than those corresponding to the tetrahedral localization of copper atoms. The character of pDOS distribution in the conduction band remains almost unchanged, and the major contribution is due to chromium states. The band gap vanishes as sulfur, chromium and copper states shift to the Fermi-level region (Figure 5d).

Figure 5a,d correspond to two extreme cases of copper atoms localization. It can be assumed that the real sample structure combines simultaneously two types of copper atoms localization. The simulation of mixed both tetrahedral and octahedral copper atom localization (denoted as “(T)” and “(O)” in Figure 5b,c, respectively) allow one to observe the changes in partial DOS distribution. With an increase of number of copper atoms localized at the octahedral sites the main contribution of copper states is shifted to the Fermi level region (Figure 5b–d). This shift results the band gap narrowing across the transition from tetrahedral to octahedral copper atoms localization. The localization of one of three non-equivalent copper atoms at octahedral site leads to band gap width decreasing from 0.29 to 0.02 eV (Figure 5b). The localization of two of three non-equivalent copper atoms at octahedral sites leads to band gap vanishing (Figure 5c).

Thus, the localization of copper atoms at the octahedral *o*-sites in the ODT temperature region corresponds to the metal-insulator transition (MIT). Thus, we conclude that the order-to-disorder phase transition in CuCrS_2_ led to the MIT. Note that the cationic substitution in CuCrS_2_ lead to the MIT and significantly diminished the Seebeck coefficient [11]. Hence, the S(T) inflection feature is explained by the fact that the electronic structure is changed during the MIT. The band gap vanishing leads to the formation of metallic conductivity and correlates with electrical resistivity values which decrease in the ODT temperature region.

## 4. Conclusions

A comprehensive study of the CuCrS_2_ Seebeck coefficient and electrical resistivity in a wide temperature range of 100–740 K was carried out. It was established that the value of the Seebeck coefficient is significantly affected by the sample treatment procedure. The decrease of the Seebeck coefficient in the high-temperature region (T > 700 K) is caused by the electronic structure reconfiguration as a result of the order-disorder phase transition (ODT). The DFT calculations showed that the localization of copper atoms at the octahedral sites led to the metal-insulator transition (MIT) and a band gap vanishing. The decrease of electrical resistivity in the ODT temperature region (T > 650 K) corresponds to metallic conductivity as a result of the MIT. In the temperature region above the ODT temperature, the copper atoms are statistically distributed between tetrahedral and octahedral sites. Thus, the real sample could be considered as a mixture of both semiconductor and metallic areas. According the Anderson localization model, the electrons could be localized in the metallic areas. This results the preservation of the semiconductor conductivity character. Hence, the Seebeck coefficient and electrical resistivity do not dramatically decrease after ODT. Thus, one can observe the inflection feature regions on the Seebeck coefficient and the electrical resistivity temperature dependencies curves.

## Figures and Tables

**Table 1:** Sample treatment conditions.

Atmosphere	Annealing Temperature, °C	ρ, g/cm^3^
vacuum	650 (compressing pressure of 70 MPa)	4.07
argon	800	3.52

**Table 2:** Elemental composition of synthesized CuCrS_2_ powder and ceramic samples.

Sample	Mean Element Concentration, Mass%		
	Cu	Cr	S
Reference concentration	35	29	36
Powder	35	29	36
Vacuum treated at 650 °C	36	29	35
Argon treated at 800 °C	35	29	36

## Data Availability

The data presented in this study are available on request from the corresponding author.

## References

[1] Wolf M., Hinterding R., Feldhoff A. (2019). High power factor vs. high ZT—A review of thermoelectric materials for high-temperature application. Entropy.

[2] Liu Z., Sato N., Gao W., Yubuta K., Kawamoto N., Mitome M., Kurashima K., Owada Y., Nagase K., Lee C.-H. (2021). Demonstration of ultrahigh thermoelectric efficiency of ∼7.3% in Mg_3_Sb_2_/MgAgSb module for low-temperature energy harvesting. Joule.

[3] Manzeli S., Ovchinnikov D., Pasquier D., Yazyev O.V., Kis A. (2017). Ising pairing in superconducting NbSe_2_ atomic layers. Nat. Rev. Mater..

[4] Zhang Z., Xie Y., Peng Q., Chen Y. (2016). A theoretical prediction of super high-performance thermoelectric materials based on MoS_2_/WS_2_ hybrid nanoribbons. Sci. Rep..

[5] Chen S., Pan Y., Wang D., Deng H. (2020). Structural Stability and Electronic and Optical Properties of Bulk WS_2_ from First-Principles Investigations. J. Electron. Mater..

[6] Ataca C., Şahin H., Ciraci S. (2012). Stable, single-layer MX_2_ transition-metal oxides and dichalcogenides in a honeycomb-like structure. J. Phys. Chem. C.

[7] Lu N., Guo H., Li L., Dai J., Wang L., Mei W.N., Wu X., Zeng X.C. (2014). MoS_2_/MX_2_ heterobilayers: Bandgap engineering via tensile strain or external electrical field. Nanoscale.

[8] Bhattacharya S., Basu R., Bhatt R., Pitale S., Singh A., Aswal D.K., Gupta S.K., Navaneethan M., Hayakawa Y. (2013). CuCrSe_2_: A high performance phonon glass and electron crystal thermoelectric material. J. Mater. Chem. A.

[9] Srivastana D., Tewari G.C., Kappinen M., Nieminen R.M. (2013). First-principles study of layered antiferromagnetic CuCrX_2_ (X = S, Se and Te). J. Phys. Condens. Matter..

[10] Korotaev E.V., Peregudova N.N., Syrokvashin M.M., Mazalov L.N., Sokolov V.V., Yu I., Filatova A., Pichugin Y. (2016). Xanes of X-ray absorbtion K edges of chromium dichalcogenides CuCr_1-x_M′_x_S_2_ and MCrX_2_. J. Struct. Chem..

[11] Korotaev E.V., Syrokvashin M.M., Filatova I.Y., Pelmenev K.G., Zvereva V.V., Peregudova N.N. (2018). Seebeck Coefficient of Cation-Substituted Disulfides CuCr_1− x_Fe_x_S_2_ and Cu_1− x_Fe_x_CrS_2_. J. Electron. Mater..

[12] Korotaev E.V., Syrokvashin M.M., Filatova I.Y., Trubina S.V., Nikolenko A.D., Ivlyushkin D.V., Zavertkin P.S., Sotnikov A.V., Kriventsov V.V. (2020). XANES investigation of novel lanthanide-doped CuCr_0.99_Ln_0.01_S_2_ (Ln = La, Ce) solid solutions. Appl. Phys. A.

[13] Hansen A.L., Dankwort T., Groβ H., Etter M., König J., Duppel V., Kienle L., Bensch W. (2017). Structural properties of the thermoelectric material CuCrS_2_ and of deintercalated Cu_x_CrS_2_ on different length scales: X-ray diffraction, pair distribution function and transmission electron microscopy studies. J. Mater. Chem. C.

[14] Tewari G.C., Tripathi T.S., Kumar P., Rastogi A.K., Pasha S.K., Gupta G. (2011). Increase in the thermoelectric efficiency of the disordered phase of layered antiferromagnetic CuCrS_2_. J. Electron. Mater..

[15] Chen Y.-X., Zhang B.-P., Ge Z.-H., Shang P.-P. (2012). Preparation and thermoelectric properties of ternary superionic conductor CuCrS_2_. J. Solid State Chem..

[16] Kaltzoglou A., Vaqueiro P., Barbier T., Guilmeau E., Powell A.V. (2014). Ordered-defect sulfides as thermoelectric materials. J. Electron. Mater..

[17] Al’mukhametov R.F., Yakshibaev R.A., Gabitov E.V. (1999). Magnetic and transport properties of CuCr_1-x_ V_x_S_2_ compounds. Phys. Solid State.

[18] Al’mukhametov R.F., Yakshibaev R.A., Gabitov E.V., Abdullin A.R. (2000). Synthesis and X-ray diffraction study of CuCr_1-x_V_x_S_2_. Inorg. Mater..

[19] Al’mukhametov R.F., Yakshibaev R.A., Gabitov É.V., Abdullin A.R. (2000). Investigation of superionic phase transition in the CuCr_1-x_V_x_S_2_ system by x-ray diffraction and magnetic methods. Phys. Solid State..

[20] Al’mukhametov R.F., Yakshibaev R.A., Gabitov E.V., Abdullin A.R., Kutusheva R.M. (2003). Structural properties and ionic conductivities of CuCr_1-x_V_x_S_2_solid solutions. Phys. Stat. Sol..

[21] Akmanova G.R., Davleshina A.D. (2013). Ionic conductivity and diffusion in superionic conductors CuCrS_2_-AgCrS_2_. Lett. Mater..

[22] Engelsman F.M.R., Wiegers G.A., Jellinek F., van Laar B. (1973). Crystal structures and magnetic structures of some metal (I) chromium (III) sulfides and selenides. J. Solid State Chem..

[23] Abramova G.M., Petrakovskii G.A. (2006). Metal-insulator transition, magnetoresistance, and magnetic properties of 3d-sulfides. Low Temp. Phys..

[24] Korotaev E.V., Syrokvashin M.M., Filatova I.Y., Zvereva V.V. (2020). Vanadium doped layered copper-chromium sulfides: The correlation between the magnetic properties and XES data. Vacuum.

[25] Bhattacharya S., Bohra A., Basu R., Bhatt R., Ahmad S., Meshram K.N., Debnath A.K., Singh A., Sarkar S.K., Navneethan M. (2014). High thermoelectric performance of (AgCrSe_2_)_0.5_(CuCrSe_2_)_0.5_ nano-composites having all-scale natural hierarchical architectures. J. Mater. Chem. A.

[26] Wu D., Huang S., Feng D. (2016). Revisiting AgCrSe_2_ as a promising thermoelectric material. Phys. Chem. Chem. Phys..

[27] Vassilieva I.G., Kardash T.Y., Malakhov V.V. (2009). Phase transformations of CuCrS_2_: Structural and chemical study. J. Struct. Chem..

[28] Hong J., Delaire O. (2019). Electronic instability and anharmonicity in SnSe. Mater. Today Phys..

[29] Suwardi A., Cao J., Hu L., Wei F., Wu J., Zhao Y., Lim S.H., Yang L., Tan X.Y., Chien S.W. (2020). Tailoring the phase transition temperature to achieve high-performance cubic GeTe-based thermoelectrics. J. Mater. Chem. A..

[30] (2014). Inorganic Crystal Structure Database.

[31] BAND 2016, SCM, Theoretical Chemistry, Vrije Universiteit, Amsterdam, The Netherlands. http://www.scm.com.

[32] Murphy D.W., Chen H.S., Tell B. (1977). Superionic conduction in AgCrS_2_ and AgCrSe_2_. J. Electrochem. Soc..

[33] Thomas G.A. (1973). Critical resistivity near an order-disorder transition. Phys. Rew. Let..

[34] Korotaev E.V., Syrokvashin M.M., Peregudova N.N., Kanazhevskii V.V., Mazalov L.N., Sokolov V.V. (2015). Effects of the nearest-neighbor environment of copper atoms on the XANES spectra of layered chromium-copper disulfides. J. Struct. Chem..

[35] Le Nagard N., Collin G., Gorochov O. (1979). Etude structurale et proprietes physiques de CuCrS_2_. Mat. Res. Bull..

[36] Khumalo F.S., Huges H.P. (1980). Vacuum-ultraviolet reflectivity spectra of some α-NaFeO_2_ layer-type compounds. Phys. Rew. B.

